# Treatment of Systemic Sclerosis-Associated Interstitial Lung Disease: A Systematic Review and Network Meta-Analysis

**DOI:** 10.5152/ArchRheumatol.2025.25013

**Published:** 2025-09-01

**Authors:** Siyao Wu, Wanling Xu, Zhen Bei, Junpei Wu, Mingchun Zhang

**Affiliations:** 1 Department of Pulmonary and Critical Care Medicine, Yiwu Central Hospital, Zhejiang, China; 2 Department of Hematology, Yiwu Central Hospital, Zhejiang, China

**Keywords:** Interstitial lung disease, network meta-analysis, pharmacological treatment, systemic sclerosis

## Abstract

**Background/Aims:**

Several clinical studies have shown favorable outcomes in treating systemic sclerosis-associated interstitial lung disease (SSc-ILD), yet head-to-head comparisons regarding the efficacy and safety of these pharmacological therapies remain limited.

**Materials and Methods:**

A systematical search was conducted to identify randomized controlled trials (RCTs) on pharmacological treatments for SSc-ILD.A comprehensive systematic search was performed across Cochrane Library, Embase, PubMed, and Web of Science to identify RCTs that evaluated pharmacological interventions for SSc-ILD, specifically cyclophosphamide, mycophenolate mofetil, nintedanib, pirfenidone, tocilizumab, and rituximab. The effects of various treatments versus placebo on changes in forced vital capacity (FVC), diffusing capacity of the lungs for carbon monoxide (DLCO), and serious adverse events (SAEs) were evaluated by a Bayesian network meta-analysis. Pooled estimates, including mean difference and risk ratios with 95% CIs, were calculated to compare different therapies. The surface under the cumulative ranking probability (SUCRA) was then used to rank these therapeutic agents.

**Results:**

Tocilizumab had the highest SUCRA probability (90.4%) in slowing the deterioration of FVC. Rituximab showed the highest SUCRA probability (84.2%) in the prevention of DLCO. Moreover, rituximab showed the lowest probability (59.1%) for SAEs.

**Conclusion:**

Tocilizumab and rituximab may be the optimal interventions. Still, further direct head-to-head trials are necessary to substantiate these conclusions.

**Main Points**

- Tocilizumab and rituximab may be the optimal interventions in the treatment of systemic sclerosis-associated interstitial lung disease (SSc-ILD) among cyclophosphamide, mycophenolate mofetil, nintedanib, pirfenidone, tocilizumab, and rituximab.
- A Bayesian network meta-analysis was performed to compare the effects of different treatments with the placebo which derived from randomized controlled trials on pharmacological treatments for SSc-ILD.
- Tocilizumab and rituximab may be the optimal interventions.

## Introduction

Systemic sclerosis is a rare autoimmune disease with an unclear etiology. It is characterized by inflammation, fibrosis, and microvasculopathy. The fibrosis is primarily manifested as fibrosis of the skin and the lung.^1^ Interstitial lung disease is a common complication, occurring in about 30%-90% of patients with systemic sclerosis.^2^ The presence of interstitial lung disease signifies a poor prognosis and is the main cause of death in patients with systemic sclerosis. Data have suggested that about 35% of patients died from interstitial lung disease.^3^ Therefore, early detection and treatment of systemic sclerosis-associated interstitial lung disease (SSc-ILD) are paramount.

Currently, the pharmacological treatment of SSc-ILD remains challenging, with no consensus on standardized therapeutic approaches. Previously, cyclophosphamide was mainly used for the treatment of SSc-ILD.however, its usage was curtailed due to adverse effects such as hematuria and bone marrow suppression.^4^ The Scleroderma Lung Study II (SLS-II)^5^ found that mycophenolate mofetil matched cyclophosphamide in efficacy while causing fewer side effects, presenting a novel therapeutic option for SSc-ILD. Nonetheless, the overall therapeutic benefit remains constrained. Antifibrotic agents such as nintedanib and pirfenidone have shown considerable advancements in slowing the progression of lung function decline in idiopathic pulmonary fibrosis (IPF). The SENSCIS^6^ study found that nintedanib could also delay the decline in lung function in patients with SSc-ILD, albeit without significant improvement in lung function. There is a paucity of clinical trials on pirfenidone, and the sole existing randomized controlled trial (RCT) did not identify significant efficacy,^7^ although certain case reports have indicated some effectiveness. Currently, biological agents such as tocilizumab and rituximab are also new directions for treating SSc-ILD. The focuSSced study^8^ suggested that tocilizumab can prevent the decline in lung function in patients with SSc. Also, a multicenter open-label study indicated that rituximab is superior to conventional treatment regimens.^9^ The first double-blind RCT (DESIRES)^10^ suggested that rituximab could significantly improve lung function compared to placebo, albeit with a limited sample size.

Many systematic reviews and meta-analyses on single drugs for SSc-ILD have been conducted. However, comparative analyses between multiple drugs are relatively scarce. Recent years have seen the publication of high-quality clinical trial results for biological agents such as tocilizumab and rituximab, demonstrating promising therapeutic effects for SSc-ILD. There are only 2 meta-analyses that compared mycophenolate mofetil with cyclophosphamide,^11,12^ while 1 analysis compared mycophenolate mofetil, cyclophosphamide, and nintedanib.^13^ Another 1 compared the efficacy of immunosuppressants, antifibrotic drugs, and biological agents.^14^ However, the studies included in this meta-analysis involved the use of steroids and other drugs such as azathioprine and pomalidomide. The focuSSced and DESIRES studies were not included. Regarding the SENSCIS study that included nintedanib, there were instances where it was used in combination with mycophenolate mofetil, but these were not further differentiated.

There is currently no definitive guideline consensus regarding the optimal pharmacological treatment for SSc-ILD. Therefore, the purpose of this research was to perform a network meta-analysis (NMA) evaluating both efficacy and safety profiles of different medications used to treat SSc-ILD, based on data from RCTs.

## Methods

### Study Registration

The study adhered to the Preferred Reporting Items for Systematic Reviews and Network Meta-Analyses (PRISMA-NMA) guidelines and was prospectively registered with PROSPERO (No: CRD42024522128).

### Eligibility Criteria

#### Inclusion Criteria:

Population (P): Patients who met the 1980 or 2013 American College of Rheumatology–European League Against Rheumatism classification criteria for systemic sclerosis, whether limited systemic sclerosis (lcSSc) or diffuse systemic sclerosis (dcSSc), with ILD confirmed by high-resolution computed tomography (HRCT). No restrictions were set on race, nationality, sex, age, or disease course.

Intervention/exposure (I/E): Cyclophosphamide, mycophenolate mofetil, nintedanib, pirfenidone, tocilizumab, rituximab, monotherapy, or combination therapy.

Comparison (C): Placebo.

Outcome (O): Forced vital capacity (FVC)% predicted, diffusing capacity of the lungs for carbon monoxide (DLCO)% predicted, and serious adverse events (SAEs).

Study design (S): RCTs.

#### Exclusion Criteria:

Population (P): Patients with pulmonary function indicating FVC less than 45% of the predicted, DLCO less than 40% of the predicted, or with severe pulmonary hypertension.

Intervention/Exposure (I/E): Use or combination use of drugs outside the scope of the study.

Comparison (C): None.

Outcome (O): None.

Study design (S): Observational studies; retrospective studies; case reports.

### Data Sources and Search Strategy

A systematic search was conducted to identify RCTs on drug treatments for SSc-ILD in Cochrane Library, Embase, PubMed, and Web of Science as of December 21, 2023. Searches were conducted using MeSH terms and free-text terms, including systemic sclerosis, scleroderma, systemic, scleroderma, diffuse, lung diseases, interstitial, interstitial lung disease, cyclophosphamide, mycophenolate mofetil, nintedanib, pirfenidone, tocilizumab, and rituximab (search strategy is outlined in Supplementary Table 1).

### Study Selection

The retrieved literature was imported into End Note21. Duplicates were removed using both automated and manual identification methods. To filter out ineligible articles, the titles and abstracts of the remaining publications were screened. Full texts of the eligible articles were downloaded and further screened to identify original research suitable for the systematic review. The literature screening process was independently conducted and subjected to cross-checking by 2 researchers. A third researcher contributed to discussions when discrepancies occurred, helping the team arrive at a final decision.

### Data Extraction

The study data were independently collected by 2 researchers employing a data extraction form containing all required elements. The information included: 1. Basic information: Title, first author, year, type of study, intervention measures, duration of treatment, and outcome measures. 2. Demographics: Sample size, age, sex. Disagreements during this process were resolved by discussion between the reviewers.

### Assessment of Study Quality/Risk of Bias in Studies

Two researchers assessed the risk of bias using the RoB 2.0 version (Cochrane; London, UK) of the Cochrane risk of bias tool.^15^ Five domains comprised the assessment tool: randomization process, deviations from intended interventions, missing outcome data, measurement of the outcome, and selection of the reported results. Ratings of low risk, high risk, or unclear were assigned to each domain. A study was considered to have a low risk of bias when all domains were classified as low risk. Studies were classified as having some concerns when at least 1 domain was noted to have some issues but none were at a high risk. A study received a high risk of bias classification if it had at least 1 domain rated as high risk or multiple domains marked as having some concerns.

### Outcomes

The primary outcome measures included the FVC% predicted, DLCO% predicted, and SAEs.

### Synthesis Methods

Data analysis was performed using R version 4.3.2 (R Foundation for Statistical Computing; Vienna, Austria). A Bayesian random-effects model was utilized to compare the effects between interventions and assess the efficacy of various treatment modalities. Continuous data were analyzed using mean differences (MDs), and binary data using risk ratios (RR), both with 95% CIs. Modeling was based on a Markov chain Monte Carlo method, where 4 Markov chains ran in parallel with annealing iterations fixed at 2000. After 50 000 simulation iterations, the modeling process concluded. Model fit and overall consistency comparisons were performed using the deviance information criterion. For closed-loop networks, local consistency was evaluated through node-splitting analysis. Forest plots comparing the efficacy of different interventions were generated, with placebo serving as the reference standard for comparison. Additionally, interventions were subjected to ranking based on the surface under the cumulative ranking curve (SUCRA). Treatment effect differences among interventions were compared using a league table. The limited study count prevented the creation of a funnel plot for publication bias analysis.

## Results

### Study Selection

**Figure 1.**
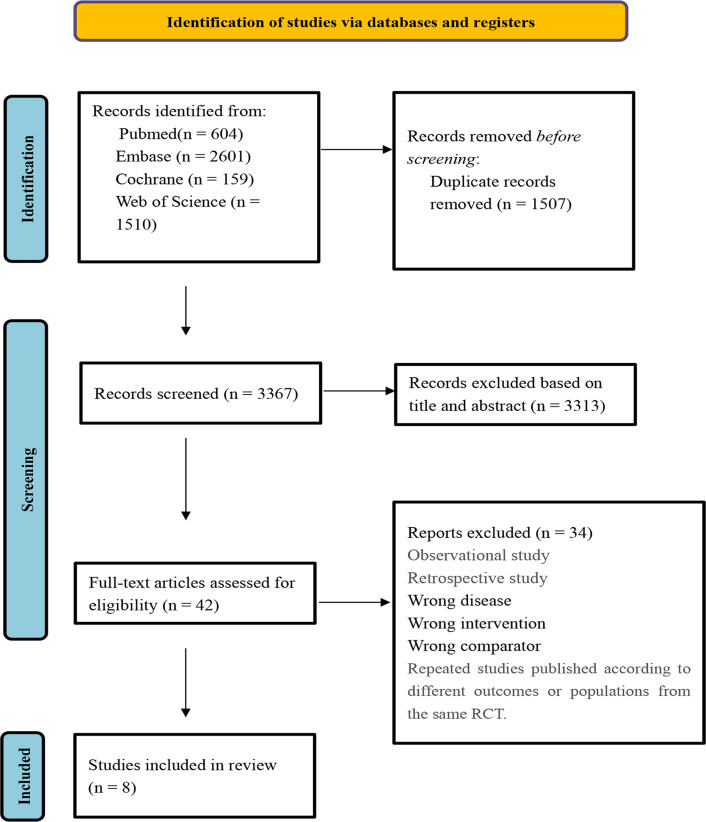
Literature screening process.

A preliminary search yielded 4874 relevant articles. After excluding 1507 duplicates, 42 articles were retained following title and abstract screening. Subsequently, 42 articles were subjected to full-text screening, resulting in the inclusion of 8 studies.^4,5,7,8,^The process of literature screening is illustrated in Figure 1.

### Study Characteristics

A total of 8 studies involving a total of 1195 patients were included in this study, with authors from the United States, India, and Japan. The studies spanned from 2006 to 2021, with the majority being published in the last 5 years. The 1980/2013 American College of Rheumatology–European League Against Rheumatism classification criteria for systemic sclerosis were primarily utilized, with interstitial lung disease confirmed through chest CT. The pharmacological interventions in the studies included cyclophosphamide, mycophenolate mofetil, nintedanib, pirfenidone, tocilizumab, and rituximab. The basic characteristics of the included studies are provided in Table 1.

### Assessment of Study Quality/Risk of Bias in Studies

**Figure 2.**
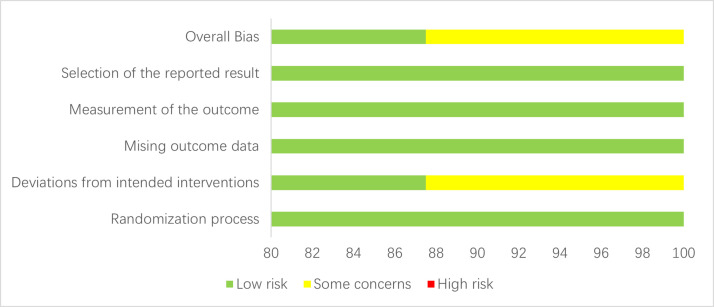
Risk of bias graph.

Regarding randomization methods, 3 studies^7,10,18^ used a computer-generated random sequence, 2 studies^4,8^ employed a permuted-block design, 1 study^16^ used a pseudo-random number generator, 1 study^5^ utilized a center-blocked design, and 1 study^17^ only mentioned being randomized in a 1 : 1 ratio in blocks of 10 to the 2 study groups. In terms of allocation concealment, all 8 studies used allocation concealment. Regarding blinding, 7 studies were blinded, while only 1 study^18^ did not employ blinding. The data from all studies were complete, with no evidence of selective reporting found. The risk of bias results for the included studies are presented in Figures 2 and Supplementary Figure 1.

## Meta-Analysis

### Forced Vital Capacity% Predicted

1. **Association between interventions**

Eight studies^4,5,7,8,10,16-18^ reported the FVC% predicted, involving 6 drugs: cyclophosphamide, mycophenolate mofetil, nintedanib, pirfenidone, tocilizumab, and rituximab. Two studies reported pairwise comparisons between drugs, 5 studies reported comparisons of drugs with placebo, and 1 study reported pairwise comparisons of drugs with placebo. Among these, most studies were related to mycophenolate mofetil, and the graphic displayed a closed loop, as shown in Supplementary Figure 2.

**Synthesized results**

**Figure 3.**
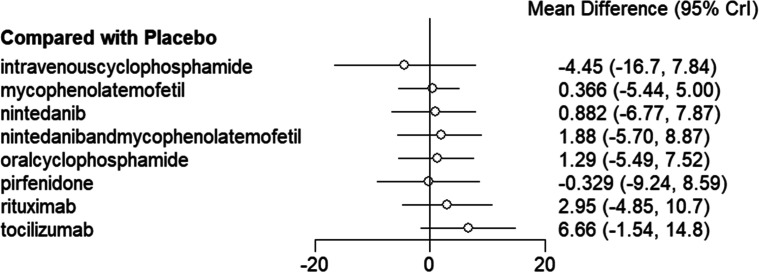
Forest plot for the meta-analysis of FVC% predicted between placebo and drugs.

Analysis of the NMA revealed no significant statistical differences (*P *> .05, *I*^2^ = 7%) between the 6 drugs compared to placebo (Figure 3), nor among the drugs themselves (*P *> .05) (Table 2). The top 3 in the SUCRA were tocilizumab (0.904), rituximab (0.709), and mycophenolate mofetil and nintedanib (0.595) (Table 5).

### Diffusing Capacity of the Lungs for Carbon Monoxide% Predicted

1. **Association between interventions**

Four studies^4,5,10,17^ reported the DLCO% predicted, involving 3 drugs: cyclophosphamide, mycophenolate mofetil, and rituximab. One study reported pairwise comparisons between drugs, and 3 studies reported comparisons of drugs with placebo. Among these, most studies were related to cyclophosphamide and mycophenolate mofetil, and the graphic displayed a closed loop, as shown in Supplementary Figure 3.

**Synthesized results**

**Figure 4.**
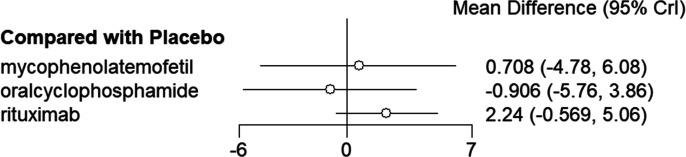
Forest plot for the meta-analysis of DLCO% predicted between placebo and drugs.

The NMA results indicated no significant statistical differences (*P *> .05, *I*^2^ = 2%) between the 3 drugs compared to placebo (Figure 4) nor among the drugs themselves (*P *> .05) (Table 3). The top 3 in the SUCRA were rituximab (0.842), mycophenolate mofetil (0.566), and placebo (0.365) (Table 5).

### Serious Adverse Events

1. **Association between interventions**

Six studies^4,5,10,16-18^ reported the incidence of SAEs, involving 4 drugs: cyclophosphamide, mycophenolate mofetil, nintedanib, rituximab. Two studies conducted pairwise comparisons of drugs; 3 studies compared drugs with placebo; and 1 study reported both comparisons. Among the 4 drugs, mycophenolate mofetil was reported most frequently, and there was closed loops, as shown in Supplementary Figure 4.

**Synthesized results**

**Figure 5.**
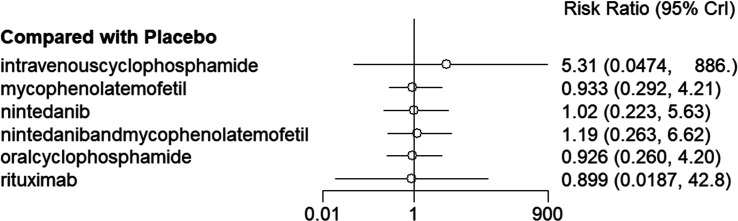
Forest plot for the meta-analysis of SAEs between placebo and drugs.

Network meta-analysis results indicated no significant statistical differences between placebo and the 4 drugs (*P *> .05, *I*^2^ = 16%) (Figure 5), and there was no significant statistical difference between the drugs (*P *> .05). (Table 4). According to the SUCRA rankings, the top 3 drugs are rituximab (0.591), oral cyclophosphamide (0.585), and mycophenolate mofetil (0.585) (Table 5).

## Discussion

### Summary of Evidence

This study included 8 RCTs involving 6 drugs (cyclophosphamide, mycophenolate mofetil, nintedanib, pirfenidone, tocilizumab, rituximab) and placebo, totaling 1195 patients with SSc-ILD. In terms of improving lung function, the 6 drugs exhibited no statistically significant variances compared to placebo, nor between-drug significant differences. In improving FVC, tocilizumab, and rituximab ranked the highest in SUCRA. In improving DLCO, rituximab and mycophenolate mofetil ranked the highest. Regarding safety, the 4 drugs similarly demonstrated no statistically significant differences compared to placebo, nor among themselves, with rituximab ranking the highest in SUCRA. Although no statistically significant differences were noted between the drugs upon comparison, from the evidence, tocilizumab may have the optimal effect in slowing the decline of FVC, rituximab may be most effective in improving DLCO, and it demonstrated the optimal safety profile in terms of SAEs.

### Evidence Analysis

Tocilizumab is a recombinant humanized monoclonal antibody against the human interleukin-6 (IL-6) receptor. It is traditionally utilized for treating rheumatoid arthritis. Early studies found that blood IL-6 levels were increased in patients with systemic sclerosis, especially in dcSSc, which was closely related to the development of skin fibrosis and interstitial pneumonia. Inhibiting the IL-6 signaling pathway using tocilizumab may reduce skin fibrosis in patients with systemic sclerosis.^19-21^ The Phase II fascinate^22^ trial suggested that tocilizumab might delay the decline in FVC% in patients with SSc-ILD. Subsequent phase III focuSSced study^8^ further validated this observation, showing a 6.4% difference in the mean change in FVC% predicted between tocilizumab and placebo in the interstitial lung disease subgroup, indicating that tocilizumab effectively preserves lung function. Therefore, the FDA has approved subcutaneous tocilizumab for slowing the rate of lung function decline in adult patients with SSc-ILD. Post hoc analyses of focuSSced showed that in SSc-ILD patients with mild (lung involvement > 5%-10%), moderate (lung involvement > 10%-20%), and severe (lung involvement > 20%) disease, as assessed by quantitative interstitial lung disease based on HRCT, the mean changes in FVC% in the TCZ group at Week 48 were −4.1, 0.7, and 2.1, vs. −10.0, −5.7, and −6.7, in the placebo group.^23^ This suggested that tocilizumab may benefit SSc-ILD patients across varying degrees of lung function impairment. Contrary to the previous approach of initiating treatment only when clinical symptoms or lung function deterioration become evident, results obtained from the focuSSced study suggested that early intervention in patients with early-stage ILD to prevent disease progression may be a viable treatment option. A meta-analysis indicated that compared to placebo, tocilizumab may slow the progression of SSc-ILD, although the level of evidence is very low.^24^ A large, propensity-score-matched, controlled observational real-life EUSTAR study^25^ did not reveal significant differences of tocilizumab in improving skin and lung function compared to other treatments. Nevertheless, the consistent direction of all predefined outcome measures raises hypotheses regarding potential efficacy in a broader SSc population. The sample size of the study is relatively small, and all the enrolled subjects were patients with early-stage dcSSc and mild lung function impairment, with the disease in an active phase. Given these limitations, further high-quality clinical trials are necessary to validate these findings.

Rituximab is a chimeric monoclonal antibody, it can specifically bind to the transmembrane antigen CD20 on B-cell surfaces and is mainly used for treating lymphoma and chronic lymphocytic leukemia. Additionally, rituximab can inhibit B cells involved in treating autoimmune diseases such as granulomatosis with polyangiitis, rheumatoid arthritis, and microscopic polyangiitis. Systemic sclerosis is also classified as an autoimmune disorder, with considerable research focused on the utilization of rituximab for treatment. Multiple studies have demonstrated that rituximab can effectively delay the decline in lung function. A multicenter, open-label, comparative study revealed that rituximab is more effective than traditional treatments such as mycophenolate mofetil. This therapy showed an increase in FVC% at 2 years of treatment.^9^ However, a prospective study from the EUSTAR indicated that after a median follow‐up of 2 years, rituximab significantly improved skin fibrosis but did not show a significant effect on lung function improvement.^26^ The NMA revealed that rituximab exhibited no significant statistical difference when compared with placebo. However, the SUCRA suggested that the effectiveness of rituximab was second only to tocilizumab, and rituximab outperformed immunosuppressive agents in improving DLCO%. Regarding the efficacy of rituximab, 2 studies were included: 1 non-blinded RCT compared rituximab with intravenous cyclophosphamide, and 1 double-blind RCT compared rituximab with placebo. Both studies indicated that rituximab has a favorable effect on delaying the decline in lung function and can even improve lung function. In the DESIRES trial,^10^ most patients had mild lung function impairment, and approximately 80% of the patients had dcSSc. There was a 2.96% difference in FVC% predicted between rituximab and placebo. In the study by Sircar et al,^18^ all enrolled patients had dcSSc and moderate lung function impairment with high modified Rodnan skin score (mRSS) scores, and rituximab was found to be more significantly effective than intravenous cyclophosphamide. Nevertheless, the number of participants was small and the follow-up duration for both studies was only 6 months.

In previous years, treatment options for SSc-ILD were limited. Based on the results of the SLS-I study, 1 year of oral cyclophosphamide in patients with SSc-ILD demonstrated a significant yet modest favorable impact on lung function. Cyclophosphamide is the most used immunosuppressant;^27^ however, its usage is restricted due to its associated side effects such as leukopenia and neutropenia. A retrospective analysis of SLS-I study indicated that the severity of reticular infiltrates on baseline HRCT and the baseline mRSS might have the ability to predict patient response to CYC (Cyclophosphamide)therapy, and CYC produced stronger effects in those with more severe skin and/or lung disease.^28^ In the CYC group, the mean FVC improvement in those with baseline FVC < 70% predicted was 4.62% at 12 months and 6.8% at 18 months, while in patients with baseline FVC > 70% predicted, the mean treatment effect was 0.55% at 12 months and 2.67% at 18 months.^29^ Therefore EULAR suggests the use of CYC for SSc-ILD, especially for patients with progressive lung disease.^30^

The SLS-II study^5^ demonstrated that a 2-year course of mycophenolate mofetil exhibited comparable efficacy to a 1-year regimen of cyclophosphamide in enhancing FVC% predicted, with fewer side effects. Of the participants, 58% had dcSSc, and the majority had moderate lung function impairment. Post hoc analyses showed significant increases from baseline in FVC% predicted in both treatment groups, not only at months 12 and 18 but also at months 21 and 24. A meta-analysis also reached the same conclusion,^11^ and mycophenolate mofetil is gradually becoming an alternative treatment to cyclophosphamide. However, the SLS-II study was constrained by certain limitations, notably the absence of a placebo-controlled comparison. An analysis incorporating data from both the SLS-I and SLS-II studies revealed notable improvements in FVC% predicted and DLCO% predicted with mycophenolate mofetil in comparison to placebo.^31^ The subgroup analysis of the SENSCIS study showed that compared to placebo, mycophenolate mofetil combined with nintedanib was the most effective in reducing the annual rate of FVC% (−0.9), but no significant heterogeneity was found when it compared to nintedanib alone.^16^ Nevertheless, this finding provides a new perspective on the potential for combination therapy in the treatment of SSc-ILD. However, Naidu et al^17^ suggested that mycophenolate mofetil had no significant effect in treating patients with mild pulmonary impairment (baseline FVC%% ≥ 70% of predicted), although the study had a small sample size and short treatment duration.

Nintedanib and pirfenidone have been approved by the FDA for treating IPF. Moreover, given the clinical and pathological similarities between IPF and SSc-ILD, considerable research efforts are currently underway to explore their therapeutic applicability in the latter condition. In the SENSCIS study,^6^ 51.9% of the patients had dcSSc, with most experiencing moderate lung function impairment. Nintedanib, when compared to placebo, decreased the annual rate of decline in FVC% by 1.2 over a 52-week period. However, it’s noteworthy that 48.4% of patients enrolled in this study were concurrently receiving mycophenolate mofetil. Subgroup analysis showed a reduction in the annual rate of decline in FVC% of 1.5 with nintedanib compared to placebo.^16^ Based on this study, nintedanib also obtained FDA approval for the treatment of SSc-ILD.

A 24-week prospective controlled cohort study suggested that pirfenidone in combination with immunosuppressants can significantly improve FVC% predicted in SSc-ILD.^32^ Acharya^7^ conducted the only RCT of pirfenidone treatment for SSc-ILD to date. However, pirfenidone failed to improve the FVC% predicted in this study. This study also had a limited participant cohort and a treatment duration of only 6 months. Subsequent clinical trials are imperative to corroborate the efficacy of pirfenidone.

In terms of safety, the NMA demonstrated that different agents did not increase the risk of SAEs in SSc-ILD patients compared to the placebo. The SUCRA suggested that rituximab may have the least possibility of inducing SAEs. One patient died of severe pulmonary hypertension after 5 months of treatment, which may be related to the disease process. One patient developed severe hypoalbuminemia, which led to the discontinuation of treatment. Cyclophosphamide was associated with a higher incidence of adverse reactions compared to mycophenolate mofetil, such as bone marrow suppression. However, the incidence of SAEs was similar between the 2 drugs. Unfortunately, no data on the safety of tocilizumab and pirfenidone were included.

Erre et al^14^ conducted an NMA of 8 interventions for the treatment of SSc-ILD, excluding the focuSSced and DESIRES studies but including pomalidomide, cyclophosphamide plus high dose prednisone followed by azathioprine. In the SENSICS study, which included nintedanib, there was co-administration with mycophenolate mofetil, but further subdivision was not conducted. The FVC% predicted, DLCO% predicted, SAEs, and mortality were studied. Compared with the placebo group, only rituximab significantly improved FVC% predicted (SMD = 1.00, 95% CI (0.39-1.61)). There was no significant advantage of other drugs compared with the placebo group. Compared with the placebo group, various drugs had no significant advantage in improving DLCO%, and no significant difference was noted in SAEs and mortality. The study indicated that rituximab, compared to placebo, exhibited no significant difference in FVC% predicted. However, data from the DESIRES study were included, which conversely suggested a significant improvement in FVC% predicted with rituximab. This inconsistency may be attributed to the larger overall participant pool in the study, although the number of participants in both rituximab studies is relatively modest. Flórez-Suárez et al^13^ conducted an NMA on various interventions for treating SSc-ILD, including nintedanib, mycophenolate mofetil, and cyclophosphamide. The analysis included outcome measures of decrease >5% FVC and decrease >10% FVC. The results showed that compared to placebo, none of the medications demonstrated a significant advantage (OR = 0.40, 95% CI (0.21 to 0.78)), (OR = 0.75, 95% CI (0.42 to 1.33)). Although the outcome measures for FVC were different, their conclusion is similar to the study’s findings.

### Strengths and Limitations

Pharmacotherapy of SSc-ILD is currently a prominent area of research, with a growing number of studies investigating various drugs. However, evidence comparing the efficacy and safety of these drugs remains limited. In this NMA, widely used drugs for treating SSc-ILD were updated to compare efficacy and safety differences among them. Moreover, strict inclusion and exclusion criteria were utilized, incorporating only RCTs. The included studies were published in high-quality journals, thereby yielding robust evidence. The study has certain limitations. First, the number of included RCTs was relatively small, and many studies had limited sample sizes, which may have affected the robustness of the data analysis. Secondly, there were differences in patient characteristics across studies, such as the degree of lung function impairment, the proportion of lcSSc and dcSSc, mRSS scores, and the extent of ILD on HRCT, which potentially impacted the robustness of the evidence. The FVC changes over time showed remarkable similarity between lcSSc and dcSSc in SSc-ILD according to some studies, which supports the inclusion of lcSSc patients in SSc-ILD trials and suggests potential benefits from anti-ILD drugs.^33^ Thirdly, owing to disparities in methods and course of treatment, meta-regression analysis could not be conducted. Certain outcome measures in the study, such as DLCO% and the incidence of SAEs, rely on a restricted number of primary articles, potentially affecting the outcomes.

Based on the evidence gleaned from this study, compared to placebo, 6 drugs (cyclophosphamide, mycophenolate mofetil, nintedanib, pirfenidone, tocilizumab, rituximab) showed no significant statistical differences in efficacy and safety. However, as per the SUCRA results, except for intravenous cyclophosphamide, the drugs mentioned above are effective in the treatment of SSc-ILD, the biologics tocilizumab and rituximab may be the optimal interventions. Given the current scarcity of head-to-head studies, and differences in the number of enrolled patients, disease subset, mRSS, lung function, HRCT, and treatment courses across various studies, the certainty of the evidence is low, therefore additional high-quality RCTs are imperative to further corroborate the efficacy and safety of these medications. For example, relevant head-to-head studies on direct comparisons of biologics with antifibrotic drugs and immunosuppressants, and even comparisons between biologics should be conducted in systemic sclerosis patients with lung function impairment at different stages and different disease subsets.

## Supplementary Materials

Supplementary Material

## Figures and Tables

**Table 1.** The Basic Characteristics of the Included Studies

Author/Year	Design	Number of Subjects	Therapeutic Drug/Control	Intervention	Treatment Course	Primary Outcome Measures	Secondary Outcome Measures
D.P. Tashkin, 2006^4^	RCT	158	Cyclophosphamide/placebo	Oral 1-2 mg/kg/day	1 year	FVC%	TLC%, DLCO%, DL:VA, HAQ-DI, SF-36
D.P. Tashkin, 2016^5^	RCT	142	Cyclophosphamide/mycophenolate	Oral 1.8-2.3 mg/kg/day Oral 0.5-1·5 g twice/day	1 year/2 years	FVC%	DLCO%, TDI, mRSS, quantitative HRCT scores
N. Acharya, 2020^7^	RCT	34	Pirfenidone/placebo	Oral 200-800 mg thrice/day	6 months	Improved/stabilized in FVC	FVC%
D. Khanna, 2020^9^	RCT	136	Tocilizumab/placebo	Subcutaneous 162 mg weekly	48 weeks	mRSS	FVC%, time to treatment failure, patient-reported, and physician-reported outcomes
G. Naidu, 2020^18^	RCT	41	Mycophenolate/placebo	Oral 0.5-1 g twice/day	6 months	FVC%	SF36v2, MDI, FVC in ATA(+), adverse events
S. Ebata, 2021^11^	RCT	48	Rituximab/placebo	Intravenous 375 mg/m^2^ per week	4 weeks	mRSS	FVC%, DLCO%, TLC, SF-36, HAQ-DI
G. Sircar, 2018^19^	RCT	60	Rituximab/IV cyclophosphamide	Intravenous 1000 mg at 0 and 15 days Intravenous 500 mg/m^2^ every 4 weeks	24 weeks	FVC%	FVC-I, mRSS, 6MWD, Medsgers score, pulmonary hypertension
K.B. Highland, 2021^17^	RCT	576	Nintedanib/mycophenolate/ Nintedanib + mycophenolate /placebo	Oral 150 mg twice/day 2g/day	52 weeks	Annual rate of decline in FVC (milliliters per year)	SGRQ, mRSS

RCT, randomized controlled trial; FVC%, percentage of predicted forced vital capacity; DLCO%, percentage of predicted diffusing capacity of the lungs for carbon monoxide; TLC%, percentage of predicted total lung capacity; DL:VA, the diffusing capacity adjusted for alveolar volume; SF-36, the Medical Outcomes Study 36-item Short-Form General Health Survey; HAQ-DI, the disability index of the Health Assessment Questionnaire; TDI, Transition Dyspnea Index; mRSS, modified Rodnan skin score; SF36v2, Short Form-36; MDI, Mahler’s Dyspnoea Index; TLC, total lung capacity; FVC, forced vital capacity; ATA, anti-nuclear antibody; FVC-I, forced vital capacity absolute change in litres; 6MWD, 6-min walk distance; SGRQ, St George’s Respiratory Questionnaire; HRCT, high-resolution computed tomography.

**Table 2.** League Table for the Effects of Drugs for FVC% Predicted

	Intravenous Cyclophosphamide	Mycophenolate mofetil	Nintedanib	Nintedanib and Mycophenolate mofetil	Oral Cyclophosphamide	Pirfenidone	Placebo	Rituximab	Tocilizumab
Intravenous cyclophosphamide	Intravenous cyclophosphamide	4.8 (−9.15, 17.5)	5.31 (−9.32, 19.12)	6.32 (−8.35, 20.14)	5.76 (−8.55, 19.18)	4.19 (−11.06, 19.04)	4.52 (−7.89, 16.61)	7.44 (−2.25, 16.94)	11.13 (−3.72, 25.77)
Mycophenolate mofetil	−4.8 (−17.5, 9.15)	Mycophenolate mofetil	0.43 (−6.52, 8.01)	1.45 (−5.51, 9.05)	0.93 (−5.11, 7.56)	−0.58 (−10.37, 10.07)	−0.38 (−4.94, 5.43)	2.51 (−6.16, 12.55)	6.32 −2.86, 16.62)
Nintedanib	−5.31 (−19.12, 9.32)	−0.43 (−8.01, 6.52)	Nintedanib	1.01 (−6.79, 8.84)	0.46 (−8.44, 9.47)	−1.1 (−12.31, 10.51)	−0.88 (−7.75, 6.75)	2.05 (−8.16, 13.07)	5.83 (−4.82, 17.07)
Nintedanib and mycophenolate mofetil	−6.32 (−20.14, 8.35)	−1.45 (−9.05, 5.51)	−1.01 −8.84, 6.79)	Nintedanib and mycophenolate mofetil	−0.55 (−9.47, 8.46)	−2.11 (−13.34, 9.53)	−1.88 (−8.79, 5.78)	1.06 (−9.21, 12.13)	4.82 (−5.92, 16.02)
Oral cyclophosphamide	−5.76 (−19.18, 8.55)	−0.93 (−7.56, 5.11)	−0.46 (−9.47, 8.44)	0.55 (−8.46, 9.47)	Oral cyclophosphamide	−1.56 (−12.36, 9.58)	−1.29 (−7.52, 5.51)	1.65 (−8.13, 12.12)	5.39 (−4.85, 16.12)
Pirfenidone	−4.19 (−19.04, 11.06)	0.58 (−10.07, 10.37)	1.1 (−10.51, 12.31)	2.11 (−9.53, 13.34)	1.56 (−9.58, 12.36)	Pirfenidone	0.3 (−8.46, 9.22)	3.26 (−8.38, 15.11)	6.95 (−5.04, 19.06)
Placebo	−4.52 (−16.61, 7.89)	0.38 (−5.43, 4.94)	0.88 (−6.75, 7.75)	1.88 (−5.78, 8.79)	1.29 (−5.51, 7.52)	−0.3 −9.22, 8.46)	Placebo	2.96 (−4.82, 10.74)	6.66 (−1.55, 14.82)
Rituximab	−7.44 (−16.94, 2.25)	−2.51 (−12.55, 6.16)	−2.05 (−13.07, 8.16)	−1.06 (−12.13, 9.21)	−1.65 (−12.12, 8.13)	−3.26 (−15.11, 8.38)	−2.96 (−10.74, 4.82)	Rituximab	3.71 −7.6, 14.92)
Tocilizumab	−11.13 −25.77, 3.72)	−6.32 (−16.62, 2.86)	−5.83 (−17.07, 4.82)	−4.82 (−16.02, 5.92)	−5.39 (−16.12, 4.85)	−6.95 (−19.06, 5.04)	−6.66 (−14.82, 1.55)	−3.71 (−14.92, 7.6)	Tocilizumab

**Table 3.** League Table for the Effects of Drugs for DLCO% Predicted

	Mycophenolate Mofetil	Oral Cyclophosphamide	Placebo	Rituximab
Mycophenolate mofetil	Mycophenolate mofetil	−1.59 (−5.46, 2.26)	−0.71 (−6.08, 4.78)	1.54 (−4.47, 7.64)
Oral cyclophosphamide	1.59 (−2.26, 5.46)	Oral cyclophosphamide	0.91 (−3.86, 5.76)	3.15 (−2.33, 8.73)
Placebo	0.71 (−4.78, 6.08)	−0.91 (−5.76, 3.86)	Placebo	2.24 (−0.57, 5.06)
rituximab	−1.54 (−7.64, 4.47)	−3.15 (−8.73, 2.33)	−2.24 (−5.06, 0.57)	Rituximab

**Table 4.** League Table for the Effects of Drugs for SAEs

	Intravenous Cyclophosphamide	Mycophenolate Mofetil	Nintedanib	Nintedanib and Mycophenolate Mofetil	Oral Cyclophosphamide	Placebo	Rituximab
Intravenous cyclophosphamide	Intravenous cyclophosphamide	0.184 (0.001, 25.693)	0.197 (0.001, 29.467)	0.23 (0.001, 34.338)	0.179 (0.001, 25.57)	0.188 (0.001, 21.108)	0.184 (0.005, 2.386)
Mycophenolate mofetil	5.447 (0.039, 1031.099)	Mycophenolate mofetil	1.097 (0.2, 5.156)	1.281 (0.236, 6.041)	0.989 (0.239, 3.728)	1.071 (0.238, 3.425)	0.925 (0.015, 52.203)
Nintedanib	5.084 (0.034, 1049.898)	0.911 (0.194, 4.999)	Nintedanib	1.168 (0.217, 6.458)	0.904 (0.139, 6.295)	0.98 (0.178, 4.486)	0.863 (0.013, 53.785)
Nintedanib and mycophenolate mofetil	4.344 (0.029, 903.178)	0.781 (0.166, 4.229)	0.856 (0.155, 4.608)	Nintedanib and mycophenolate mofetil	0.773 (0.118, 5.296)	0.838 (0.151, 3.799)	0.735 (0.011, 46.026)
Oral cyclophosphamide	5.6 (0.039, 1065.773)	1.011 (0.268, 4.176)	1.106 (0.159, 7.182)	1.294 (0.189, 8.459)	Oral cyclophosphamide	1.08 (0.238, 3.853)	0.941 (0.015, 55.483)
Placebo	5.308 (0.047, 885.938)	0.933 (0.292, 4.21)	1.02 (0.223, 5.627)	1.193 (0.263, 6.619)	0.926 (0.26, 4.204)	Placebo	0.899 (0.019, 42.796)
Rituximab	5.446 (0.419, 200.247)	1.081 (0.019, 67.234)	1.159 (0.019, 76.708)	1.361 (0.022, 90.765)	1.062 (0.018, 66.315)	1.112 (0.023, 53.518)	Rituximab

**Table 5.** Bayesian NMA Probabilities and Rankings of Drugs in Primary Outcome Measures

Interventions	FVC%		DLCO%		SAEs	
	SUCRA	Rank	SUCRA	Rank	SUCRA	Rank
Intravenous cyclophosphamide	0.158	9	/	/	0.223	7
Oral cyclophosphamide	0.528	4	0.226	4	0.585	2
Mycophenolate mofetil	0.397	6	0.566	2	0.585	3
Nintedanib	0.469	5	/	/	0.530	5
Mycophenolate mofetil and nintedanib	0.595	3	/	/	0.439	6
Pirfenidone	0.384	7	/	/	/	/
Tocilizumab	0.904	1	/	/	/	/
Rituximab	0.709	2	0.842	1	0.591	1
Placebo	0.354	8	0.365	3	0.544	4

## Data Availability

All data generated or analyzed during this study are included in this published article and its supplementary information files.
