# Supplementary material for: Treatment of Systemic Sclerosis-Associated Interstitial Lung Disease: A Systematic Review and Network Meta-Analysis
Source: Arch Rheumatol. 2025 Sep 1;40(3):395–406. doi: 10.5152/ArchRheumatol.2025.25013 (PMC12502852; doi:10.5152/ArchRheumatol.2025.25013)
Supplement: Supplementary Material [file supplementary_material.pdf]

Supplementary Table 1. Search strategy

(((((((Cyclophosphamide[MeSH Terms]) OR (Cyclophosphamide[Title/Abstract])) OR ((Mycophenolic Acid[MeSH Terms]) OR (mycophenolate mofetil[Title/Abstract]))) OR ((Rituximab[Title/Abstract]) OR (Tocilizumab tocilizumab [Supplementary Concept]))) OR ((Nintedanib[Title/Abstract]) OR (nintedanib [Supplementary Concept]))) OR ((pirfenidone[Title/Abstract]) OR (pirfenidone [Supplementary Concept]))) OR ((Tocilizumab[Title/Abstract]) OR (tocilizumab [Supplementary Concept]))) AND ((((((((((((((((((((((((((((Lung Diseases, Interstitial[MeSH Terms]) OR (Diffuse Parenchymal Lung Disease[Title/Abstract])) OR (Interstitial Lung Diseases[Title/Abstract])) OR (Diffuse Parenchymal Lung Diseases[Title/Abstract])) OR (Interstitial Lung Disease[Title/Abstract])) OR (Lung Disease, Interstitial[Title/Abstract])) OR (Pneumonia, Interstitial[Title/Abstract])) OR (Interstitial Pneumonia[Title/Abstract])) OR (Interstitial Pneumonias[Title/Abstract])) OR (Pneumonias, Interstitial[Title/Abstract])) OR (Pneumonitis, Interstitial[Title/Abstract])) OR (Interstitial Pneumonitides[Title/Abstract])) OR (Interstitial Pneumonitis[Title/Abstract])) OR (Pneumonitides, Interstitial[Title/Abstract])) OR (interstitial lung disease[Title/Abstract])) OR (diffuse interstitial pneumopathy[Title/Abstract])) OR (diffuse parenchyma lung disease[Title/Abstract])) OR (diffuse parenchymal pulmonary disease[Title/Abstract])) OR (diffuse parenchymal pulmonary disorder[Title/Abstract])) OR (interstitial lung disorder[Title/Abstract])) OR (interstitial pneumopathy[Title/Abstract])) OR (interstitial pulmonary disease[Title/Abstract])) OR (interstitial pulmonary disorder[Title/Abstract])) OR (lung diseases, interstitial[Title/Abstract])) OR (pneumopathy, interstitial[Title/Abstract])) OR (interstitial lung disease[Title/Abstract])) OR (Lung Diseases, Interstitial[Title/Abstract])) AND (((((((((((((((((((((((((((((((((Scleroderma, Diffuse[MeSH Terms]) OR (Scleroderma, Systemic[MeSH Terms])) OR (Scleroderma, Systemic[Title/Abstract])) OR (Scleroderma, Diffuse[Title/Abstract])) OR (Systemic Sclerosis[Title/Abstract])) OR (Sclerosis, Systemic[Title/Abstract])) OR (Systemic Scleroderma[Title/Abstract])) OR (Scleroderma, Progressive[Title/Abstract])) OR (Progressive Scleroderma[Title/Abstract])) OR (Diffuse Cutaneous Systemic Sclerosis[Title/Abstract])) OR (Sudden Onset Scleroderma[Title/Abstract])) OR (Scleroderma, Sudden Onset[Title/Abstract])) OR (Sclerodermas, Sudden Onset[Title/Abstract])) OR (Sudden Onset Sclerodermas[Title/Abstract])) OR (Diffuse Systemic Sclerosis[Title/Abstract])) OR (Diffuse Systemic Scleroses[Title/Abstract])) OR (Scleroses, Diffuse Systemic[Title/Abstract])) OR (Sclerosis, Diffuse Systemic[Title/Abstract])) OR (Systemic Scleroses, Diffuse[Title/Abstract])) OR (Systemic Sclerosis, Diffuse[Title/Abstract])) OR (Diffuse Scleroderma[Title/Abstract])) OR (Sclerosis, Progressive Systemic[Title/Abstract])) OR (Progressive Systemic Sclerosis[Title/Abstract])) OR (Systemic Sclerosis, Progressive[Title/Abstract])) OR (generalised scleroderma[Title/Abstract])) OR (generalised scleroderma[Title/Abstract])) OR (progressive sclerodermia[Title/Abstract])) OR (progressive sclerosis, systemic[Title/Abstract])) OR (scleroderma, generalized[Title/Abstract])) OR (scleroderma, generalized[Title/Abstract])) OR (scleroderma, systemic[Title/Abstract])) OR (sclerosis, systemic progressive[Title/Abstract])) OR (systemic progressive sclerosis[Title/Abstract])))

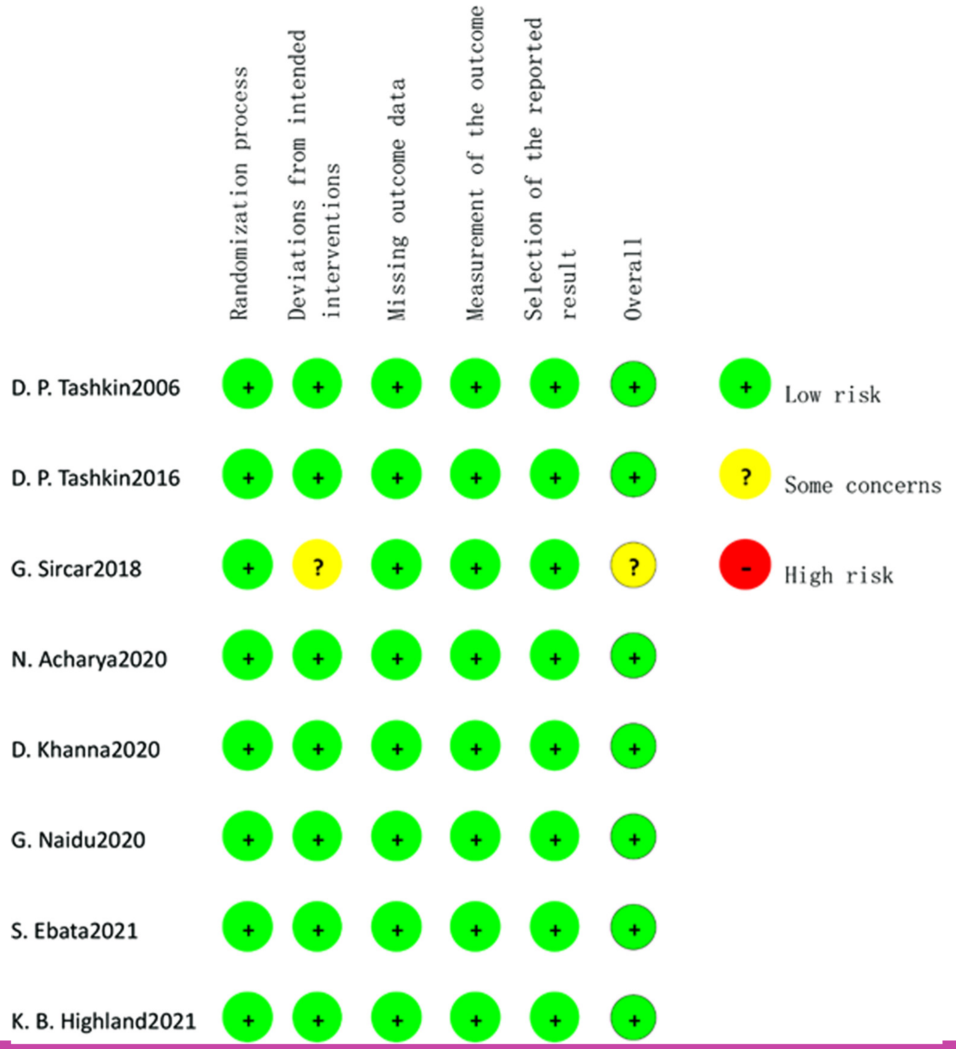

Supplementary Figure 1. Risk of bias summary.

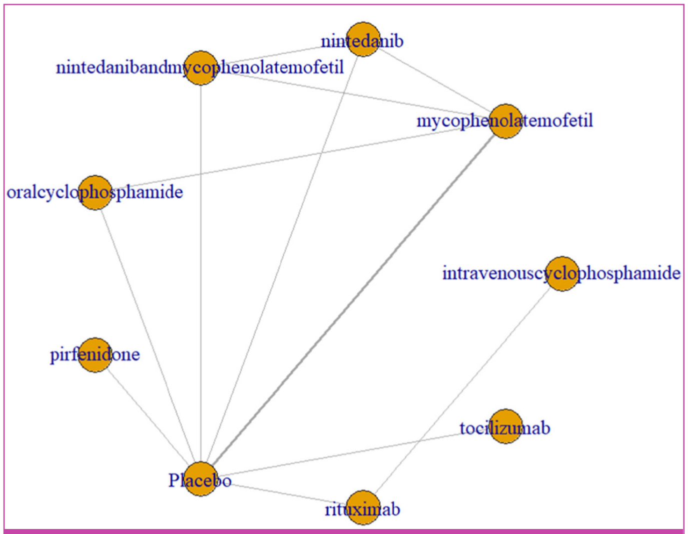

Supplementary Figure 2. Network diagram for the treatment effects of drugs for FVC% predicted in patients with SSc-ILD.

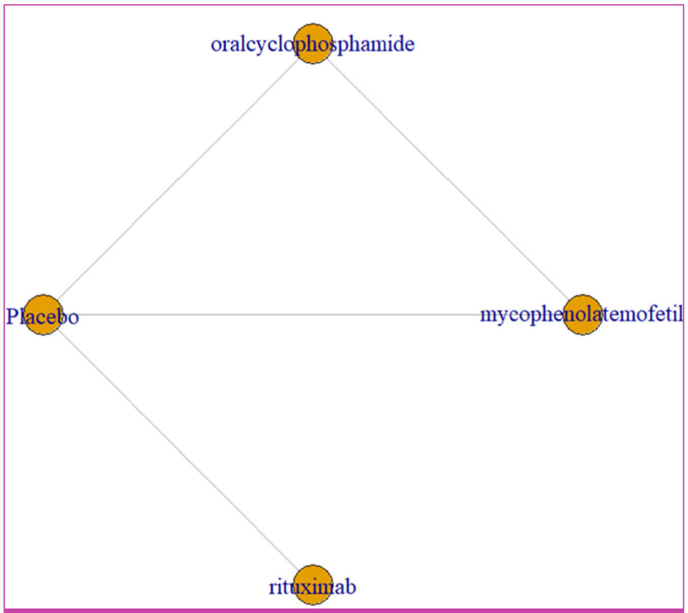

Supplementary Figure 3. Network diagram for the treatment effects of drugs for DLCO% predicted in patients with SSc-ILD.

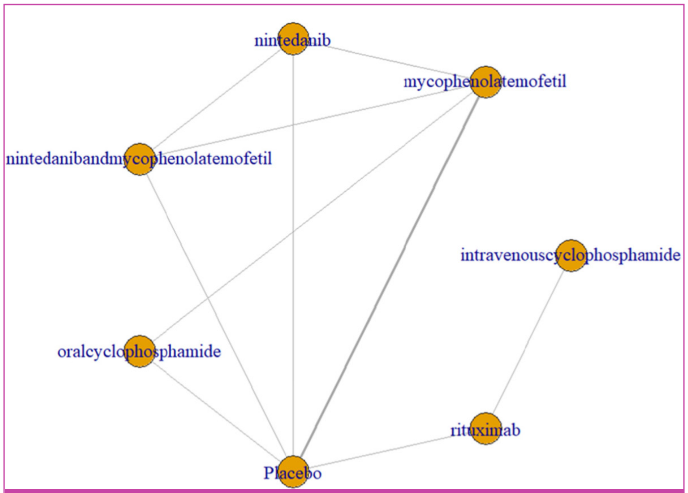

Supplementary Figure 4. Network diagram for SAEs in the treatment of SSc-ILD with drugs.
